# Inhibition of Drp1 mitochondrial translocation provides neural protection in dopaminergic system in a Parkinson’s disease model induced by MPTP

**DOI:** 10.1038/srep32656

**Published:** 2016-09-13

**Authors:** Emily Filichia, Barry Hoffer, Xin Qi, Yu Luo

**Affiliations:** 1 Department of Neurological Surgery, Case Western Reserve University, Cleveland, USA; 2 Department of Physiology & Biophysics, Case Western Reserve University, Cleveland, USA

## Abstract

Accumulating evidence suggest mitochondria-mediated pathways play an important role in dopaminergic neuronal cell death in Parkinson’s disease (PD). Drp1, a key regulator of mitochondrial fission, has been shown to be activated and translocated to mitochondria under stress, leading to excessive mitochondria fission and dopaminergic neuronal death *in vitro*. However, whether Drp1 inhibition can lead to long term stable preservation of dopaminergic neurons in PD-related mouse models remains unknown. In this study, using a classical MPTP animal PD model, we showed for the first time Drp1 activation and mitochondrial translocation *in vivo* after MPTP administration. Inhibition of Drp1 activation by a selective peptide inhibitor P110, blocked MPTP-induced Drp1 mitochondrial translocation and attenuated dopaminergic neuronal loss, dopaminergic nerve terminal damage and behavioral deficits caused by MPTP. MPTP-induced microglial activation and astrogliosis were not affected by P110 treatment. Instead, inhibition of Drp1 mitochondrial translocation diminished MPTP-induced p53, BAX and PUMA mitochondrial translocation. This study demonstrates that inhibition of Drp1 hyperactivation by a Drp1 peptide inhibitor P110 is neuroprotective in a MPTP animal model. Our data also suggest that the protective effects of P110 treatment might be mediated by inhibiting the p53 mediated apoptotic pathways in neurons through inhibition of Drp1-dependent p53 mitochondrial translocation.

Parkinson disease (PD) is the second most common neurodegenerative disorder, and the most common neurodegenerative movement disorder. Pathologically, it is characterized by the loss of pigmented dopaminergic neurons in the substantia nigra (SN) in the midbrain and the presence of proteinaceous cytoplasmic inclusions called Lewy bodies^1^. Although a number of drugs improve the symptoms, the progression of this disease is unaffected by current treatments. The etiology underlying the disease is still not clear.

Studies suggest an important role of mitochondria-dependent apoptotic pathways in the degeneration of dopaminergic neurons in PD^2^,^3^. Evidence further shows that mitochondrial fission has significant influences on mitochondrial stress responses and mitochondria-associated apoptosis^4^,^5^,^6^,^7^. Mitochondrial fission is controlled by the dynamin-related protein 1 (Drp1)^8^,^9^, which is a member of the dynamin family of large GTPase. Drp1 is primarily found in the cytosol, but it translocates from the cytosol to the mitochondria in response to various cellular stimuli to initiate the division of mitochondrial membranes through GTP hydrolysis^10^,^11^. Under stressed conditions, Drp1 translocates to the mitochondria where it triggers mitochondrial fragmentation and subsequently leads to mitochondrial depolarization. As a result, the pro-apoptotic protein Bax translocates from the cytosol to the mitochondrial outer membrane, the process of which results in the opening of the permeability transition pore and release of cytochrome c, leading in turn to intrinsic apoptotic cell death^12^,^13^. Inhibition of Drp1 by Drp1 siRNA, a dominant negative mutant Drp1 K38A, or pharmacological inhibitors reduced Bax translocation to the mitochondria and apoptotic cell death in response to various stimuli, such as UV radiation^14^, neurotoxicity^15^, glucose/oxygen deprivation^16^ and nitrosative stress^12^. Interestingly, Drp1 failed to induce apoptosis in Bax-deficient cells exposed to irradiation^14^, suggesting that Drp1-induced apoptosis is dependent on Bax.

p53 is a known stress gene implicated in programmed cell death pathways via transcription-dependent and -independent mechanisms^17^,^18^. Upon stress, a cytoplasmic pool of p53 mainly translocates to the mitochondria, an event that precedes its effect on nuclear functions^19^,^20^,^21^,^22^. Accumulation of p53 on the mitochondrial outer membrane acts as a BH3-only protein and interacts with pro-apoptotic proteins such as Bax and PUMA (p53 upregulated modulator of apoptosis), leading to apoptosis^18^,^19^. We recently showed that Drp1 binds to p53 and is required for p53 translocation to the mitochondria in models of brain ischemia^23^ and Huntington’s disease^24^. Similarly, Park *et al*. reported that Drp1 is required for p53 translocation to the mitochondria under chlorpyrifos-induced oxidative stress^25^. Other studies reported that p53 promotes Drp1-dependent mitochondrial fragmentation via direct transcriptional regulation of Drp1^26^ or transcriptional suppression of miR-499^27^. These lines of evidence indicate that there is a connection between Drp1 and p53 on mitochondrial dysfunction. However, whether Drp1 and p53 cooperate to regulate mitochondria-dependent apoptotic signals under diseased conditions, such as PD, remains unknown.

Evidence from toxin-induced dopaminergic neuronal death *in vitro* and *in vivo* supports a role for Drp1 hyperactivity and mitochondrial fission/fusion in the pathogenesis of dopaminergic neuronal death. The dopaminergic neurotoxins, 6-hydroxy dopamine (6-OHDA), rotenone, and 1-methyl-4-phenyl pyridinium (MPP^+^) all induce Drp1 hyperactivity, mitochondrial fragmentation (fission), leading to dopaminergic cell death in neuronal cultures^28^,^29^,^30^. Genetic inhibition of pro-fission Drp1 or overexpression of pro-fusion protein mitofusin-1 (Mfn1) prevents both neurotoxin-induced mitochondrial fission and neuronal cell death^28^,^29^,^30^. Further, an increase in Drp1 protein level in rotenone-induced PD in rat was recently reported^31^. Inhibition of Drp1 by a small molecule, Mdivi-1, has been shown to reduce MPTP-induced neurotoxicity in mice^32^. These lines of evidence suggest that inhibition of Drp1 hyperactivity and mitochondrial fission might be protective for dopaminergic neurons in PD. However, whether Drp1 hyperactivity and mitochondrial translocation is induced in animal models of PD has not been previously examined.

We recently developed a peptide inhibitor P110 which is rationally designed to selectively inhibit Mitochondrial fission 1 protein (Fis1)/Drp1 interaction under stressed conditions^15^. We have demonstrated that the efficacy of P110 requires the presence of Drp1. Treatment with P110 abolished Drp1 translocation to mitochondria and Drp1 polymerization under various conditions *in vitro* and *in vivo* without affecting Drp1 levels, mitochondrial structure and mitochondrial function under basal conditions^15^,^23^,^24^,^33^,^34^. We further showed that treatment with P110 reduced mitochondrial damage and organ injury in animal models of Huntington’s disease^24^, brain ischemic injury^23^ and myocardial infarction^34^. Notably, P110 treatment had no significant effects on all animal organs and blood cells evaluated histologically, and the treatment also had no effects on animal behavioral status and survival rate of naïve mice^24^,^34^,^35^. These characteristics of P110 make it a unique and specific inhibitor to modulate Drp1 activation under pathological conditions without affecting Drp1 physiological function.

In this study, using the classic subacute MPTP model of PD, we examined Drp1 translocation to mitochondria *in vivo* after MPTP administration. We showed that P110 treatment completely blocked MPTP-induced Drp1 mitochondrial translocation both in SN and striatum. In addition, we examined the effects of P110 treatment on long term and stable dopaminergic neuronal damage (30 days after MPTP administration) and the effects of P110 on mitochondrial apoptotic signaling, astrogliosis and microglial activation induced by MPTP administration.

## Methods

### Animals and treatment

All animal protocols were conducted under National Institutes Health (NIH) Guidelines using the NIH handbook *Animals in Research* and were approved by the Institutional Animal Care and Use Committee of Case Western Reserve University. The mice were housed in the animal facility of Case Western Reserve University on a 12-h light/dark diurnal cycle. Food was provided *ad libitum.*

A “subacute model” of MPTP administration regimen in mice (C57BL/6 male, 9–10 week old) was used in this study, in which MPTP was given in 7 doses (20 mg/kg, i.p) over 5 days with first 5 doses at 12 hour intervals and the last 2 doses at 24 hour intervals. At 24 or 48 hours after the first MPTP injection, brain tissues were harvested for western blot analysis and immunostaining of reactive astrocytes and microglial cells. At 1 week and 4 weeks after the last MPTP injection, mice were subjected to locomotor activity measurements. 30 days after MPTP injection, mice were perfused with 4% paraformaldehyde (PFA) and the brains were harvested for immunohistochemistry analysis. Control mice received saline injections instead of MPTP injections.

### Drp1 peptide inhibitor P110 treatment

The Drp1 peptide inhibitor P110 and control peptide TAT were synthesized by American Peptide Company (now called Bachem Americas Inc., Torrance, CA, USA) (Product # 368000, Lot # 1311151T). As previously described^15^,^24^, the peptides are synthesized as one polypeptide with TAT_47–57_ carrier in the following order: N-terminus–TAT–spacer (Gly-Gly)–cargo (Drp1_49–55_)–C-terminus. The C-termini of the peptides are modified to C(O)-NH2 using Rink Amide AM resin to increase stability. Peptides are analyzed by analytical reverse-phase high-pressure liquid chromatography (RP-HPLC) and matrix-assisted laser desorption/ionization (MALDI) mass spectrometry (MS) and purified by preparative RP-HPLC. The purity of peptides is >90% measured by RP-HPLC Chromatogram. Lyophilized peptides are stored at −80 °C freezer and are dissolved in sterile water before use.

C57BL/6 mice were implanted with a 28-day Alzet osmotic pump (model 2004, duration: 4 weeks, Alzet, Cupertino CA) containing TAT control peptide or P110-TAT peptide, which delivered peptides to the mice at a dosage of 1.5 mg/Kg/day. In our previous study we found that P110 treatment in the dosage of 1–3 mg/kg range was effective in a Huntington’s disease mouse model^24^; therefore, we tested several dosages of P110 (0.5, 1.0 and 1.5 mg/kg/day) in the MPTP mouse model and found that P110 at 1.5 mg/kg/day provided optimal protection. In the present study, we used P110 at 1.5 mg/kg/day. The pump was implanted subcutaneously in the back of 10-week old mice between the shoulders 16 hours before the first MPTP injection.

### Immunostaining

Animals were anesthetized and perfused transcardially with saline followed by 4% PFA in phosphate buffer (PB; 0.1 M; pH 7.2) at 48 h or 30 days after MPTP injections. The brains were removed, dissected, postfixed in PFA for 16 hours, and transferred to 20% and 30% sucrose in 0.1 M PB sequentially. Serial sections of the entire brain were sliced at 30 or 40 μm thickness in a cryostat. One series from every 4th section was stained for each antibody used. In order to control for staining variability, specimens from all experimental groups were included in every batch and reacted together in a net well tray under the same conditions. Sections were rinsed in 0.1 M PB, and blocked with 4% bovine serum albumin (BSA) and 0.3% Triton x-100 in 0.1 M PB. Sections were then incubated in a primary antibody solution of rabbit anti-TH (tyrosine hydroxylase) (1:1000, Chemicon, Temecula, CA) diluted in 4% BSA and 0.3% Triton x-100 in 0.1 M PB for 24 hours at 4 °C. Sections were rinsed in 0.1 M PB and incubated in biotinylated goat anti-rabbit IgG in the buffer (1:200; Vector Laboratories, Burlingame CA) for 1 hour, followed by incubation for 1 hour with avidin-biotin-horseradish peroxidase complex. Staining was developed with 2, 3′ diaminobenzidine tetrahydrochloride (0.5 mg/mL in 50 mM Tris-HCl buffer 7.4). Control sections were incubated without primary antibody. Sections were mounted on slides, and cover slipped. Histological images were acquired using an Infinity3 camera and NIKON 80i microscope. TH immunoreactivity in striatum was visualized with the use of a Nikon super-coolscan 9000 scanner. The optical density of TH immunoreactivity in striatum was analyzed using Scion Image (ver 4.02) and averaged from 3 sections with a visualized anterior commissure (AP: +0.26 mm, +0.14 mm, +0.02 mm to bregma), as previously described^36^. Observers who were blinded to the experimental groups performed all immunohistochemical measurements. Slight variations in background staining were corrected by subtracting background density of cortical regions from striatal density measurements.

For analysis of activated astrocytes and microglia, brain sections were stained with mouse anti- glial fibrillary acidic protein (GFAP) (1:500, Sigma Aldrich) and rabbit anti- Ionized calcium binding adaptor molecule 1 (Iba1) (1:1000, Wako) followed by incubation with diluted secondary antibody prepared with blocking solution (goat anti-mouse 568, 1:1000; goat anti-rabbit 480, 1:1000, Life Technologies). The slides were then washed with 0.1% Triton-X100 in TBS (tris buffered saline) and coverslipped. Images were acquired using an Olympus fluorescent microscope. Omission of the primary or secondary antibodies resulted in no staining and served as negative controls. Total number of GFAP positive cells and the diameter of Iba1 positive cells in the striatal area was quantified using Nikon NIS-Elements software and was averaged from 3 sections for each animal, as previously described^37^.

To examine Drp1 mitochondrial localization, 5 μm thick frozen sections were immunostained against sheep anti-TH (1:1000, Millipore); rabbit anti-Tom20 (1: 500, Santa Cruz Biotechnology); and mouse anti-Drp1 (1: 250, BD Bioscience) and corresponding fluorescent secondary antibodies. Coverslips were mounted and slides were imaged by confocal microscopy (Fluoview FV1000, Olympus). Pearson’s co-efficient was calculated using NIH Image J software to quantitate the co-localization between Drp1 and Tom20 in TH positive neurons as described previously^33^. At least 20 TH positive neurons in each group were counted.

### Stereologic Analysis

Unbiased stereological counts of TH-positive (TH+) neurons within the substantia nigra pars compacta (SNpc) were performed using stereological principles and analyzed with StereoInvestigator software (Microbrightfield, Williston, VT), as previously described^36^. Optical fractionator sampling^38^ was carried out on a Leica DM5000B microscope (Leica Microsystems, Bannockburn, IL) equipped with a motorized stage and Lucivid attachment (40X objective). Midbrain dopaminergic groups were outlined on the basis of TH immunolabelling, with reference to a coronal atlas of the mouse brain (Franklin and Paxinos^39^). For each tissue section analyzed, section thickness was assessed at each sampling site and guard zones of 2.5 μm were used at the top and bottom of each section. Pilot studies were used to determine suitable counting frame and sampling grid dimensions prior to counting. The following stereologic parameters were used in the final study: grid size, (X) 220 μm, (Y) 166 μm; counting frame, (X) 68.2 μm, (Y) 75 μm, depth was 20 μm. Gundersen coefficients of error for m = 1 were all less than 0.10. Stereologic estimations were performed with the same parameters in all the animal groups.

### Behavioral tests

The open field tests have been shown to provide reliable measures of motor function for MPTP-challenged mice^40^ and hence were used to evaluate the motor deficits in mice given MPTP and TAT or P110 treatment. Locomotion function was measured in mice before MPTP injection (pre) or 1 week and 4 weeks after MPTP injection as described previously. Spontaneous locomotor functions were examined using automated infra-red locomotor activity chambers, as previously described^41^. Locomotor function was assessed during a 24 hour period in an open field crossed by a grid of photobeams (VersaMax system, AccuScan Instruments) with free access to food and water in the chambers. Counts were taken of the number of photobeams broken during the trial at 5 min intervals, with separate measures for total horizontal, total distance travelled, total horizontal movement time and total vertical movement time (rearing) over a given period of time.

### HPLC measurements of DA and metabolites in striatum

Microdissected striatal tissues were frozen on dry ice and stored at −80 degree until analyzed for DA, 3, 4-Dihydroxyphenylacetic acid (DOPAC) and homovanillic acid (HVA) content using HPLC. Dopamine and metabolite concentrations were measured by high performance liquid chromatography (HPLC) as described previously^42^. DA turnover is calculated as (DOPAC + HVA)/DA.

### Isolation of mitochondrial-enriched fraction and lysate preparation

Brain tissue from SN and striatum were harvested and homogenized in the mitochondrial isolation buffer (250 mM sucrose, 20 mM HEPES-NaOH, pH 7.5, 10 mM KCl, 1.5 mM MgCl_2_, 1 mM EDTA, protease inhibitor cocktail, phosphatase inhibitor cocktail). The homogenates were spun at 800 g for 10 min at 4 °C and the resulting supernatants were spun at 10,000 g for 20 minutes at 4 °C. The pellets were then washed with lysis buffer and spun at 10,000 g again for 20 minutes at 4 °C. The final pellets were suspended in lysis buffer containing 1% Triton X-100 and were the mitochondrial-rich lysate fractions. The supernatants were spun at 100,000 g for 1 hour and the final supernatants were thus cytosolic fractions. The mitochondrial membrane proteins VDAC or Tom20 were used as marker and loading controls. Enolase was used as a marker and a loading control for cytosolic fractions.

### Western-blot analysis

Protein concentrations of mitochondrial fractions harvested from mouse brains were determined by Bradford assay. Thirty μg of proteins were resuspended in Laemmli buffer, loaded on SDS-PAGE and transferred onto nitrocellulose membranes. Membranes were probed with the indicated antibodies followed by visualization by ECL, and were then quantitated using NIH ImageJ software. The antibodies used in this study include Drp1 (1:2000, B&D Bioscience), p53 (1:500, Santa Cruz Biotechnology), Bax (1:1000, Proteintech Group Inc), PUMA (1:200, Proteintech Group Inc), voltage-dependent anion channel (VDAC, 1:2000, Abcam), Tom20 (1:5000, Santa Cruz Biotechnology) and Enolase (1:1000, Santa Cruz Biotechnology).

### Statistics

Statistical analysis was performed using Student’s t test, and one- or two-way analysis of variance (ANOVA), as appropriate, with Tukey post hoc tests or Bonferroni post hoc tests for repeated behavioral measurements. A p value equals to or less than 0.05 was considered significant.

## Results

### P110 treatment blocks MPTP-induced Drp1 mitochondria translocation *in vivo*

**Figure 1:**
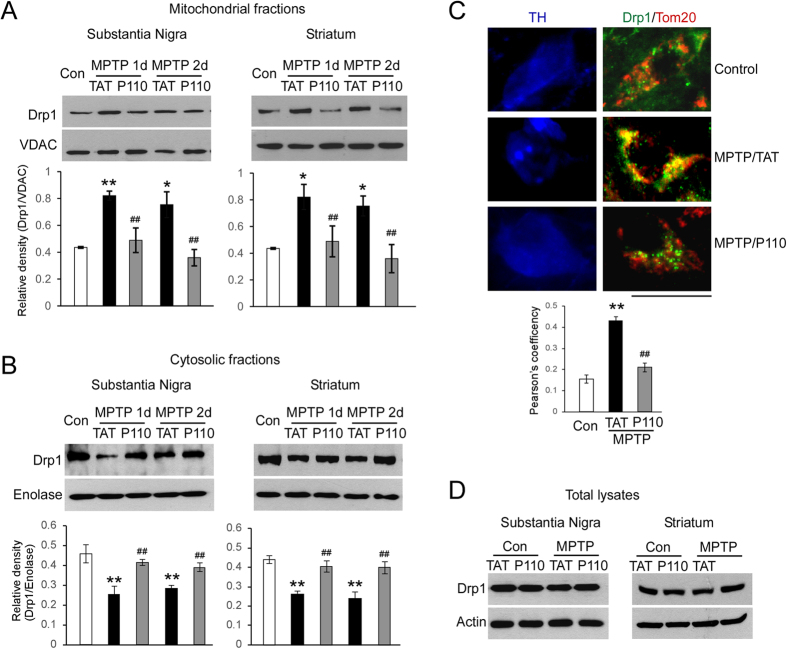
*In vivo* MPTP injection induces Drp1 translocation to mitochondria in ventral midbrain and striatum at both 1d and 2d after the first MPTP injection. (**A**) mitochondrial fractions, and (**B**) cytosolic fractions. P110 treatment (1.5 mg/kg/day) blocks Drp1 translocation to mitochondria. (**C**) confocal imaging analysis confirms co-localization of Drp1 with Tom20 in TH positive DA neurons in SNc after MPTP treatment, which is blocked by P110 treatment (more than 20 DA neurons are quantified in each group, Scale Bar = 20 μm). (**D**) Total Drp1 levels are not affected by P110 treatment. Blots are cropped to show proteins of interest. Original blots are shown in Supplemental Fig. 1. All gels have been run under the same experimental conditions. Data are expressed as mean ± SEM. *p < 0.05 and **p < 0.01 MPTP versus saline group; ^##^p < 0.01, TAT control peptide vs P110 treatment, analyzed by two-way ANOVA. (n = 3–4 in each group).

Previous studies have shown that *in vitro* treatment of dopaminergic (DA) neurons with 6-OHDA and MPP + induce Drp1 translocation to mitochondria and lead to subsequent mitochondrial fission^29^,^43^. We have demonstrated that treatment with P110 abolished Drp1 translocation to the mitochondria in dopaminergic neurons exposed to dopaminergic neruotoxins such as MPP+ 15. However, whether MPTP injections cause Drp1 mitochondrial translocation *in vivo* is unknown. In the present study, we subcutaneously treated C57BL6 mice with either peptide P110 or control peptide TAT using Alzet mini pumps (1.5 mg/Kg/day, each) 16 hours before the first MPTP injection. Drp1 levels were examined by Western blot analysis of mitochondrial and cytosolic fractions harvested from SN and striata of mice. We first showed that Drp1 translocated to mitochondria from cytosol at 24 and 48 hours after the first MPTP injection both in SN and striatum (Fig. 1A,B black bars). Treatment with P110 significantly blocked the Drp1 mitochondrial translocation when compared to that in MPTP-injected mice treated with control peptide TAT (Fig. 1A,B, gray bars). Further, confocal imaging analysis showed that Drp1 greatly co-localized with Tom20 (a marker of mitochondria) in TH-positive neurons in the SN of mice subjected to MPTP injection and that P110 treatment corrected this increased co-localization between Drp1 and Tom20 (Fig. 1C). Treatment with P110 had no effects on Drp1 total protein levels in mice treated with either vehicle or MPTP (Fig. 1D, quantification not shown, for SN, p = 0.09 saline vs MPTP; p = 0.813 TAT vs P110; for striatum, p = 0.955 saline vs MPTP; p = 0.884 TAT vs P110, ANOVA). Collectively, these results demonstrate that treatment with P110 consistently suppresses Drp1 translocation from cytosol to mitochondria in an *in vivo* animal model.

### Treatment with P110 mitigates MPTP-induced dopaminergic neurotoxicity

**Figure 2:**
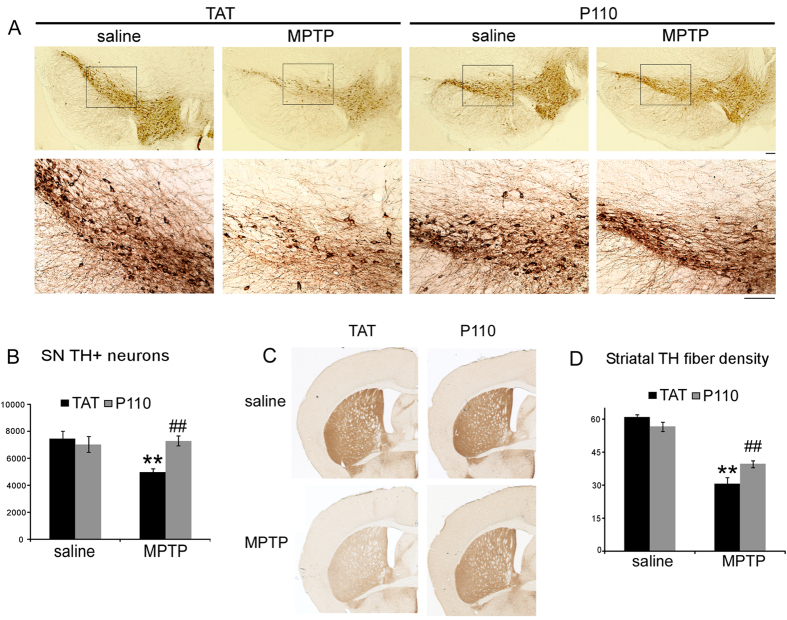
P110 (1.5 mg/kg/day) treatment results in neuroprotection in mice in a subacute MPTP model. (**A**) Representative images showing immunohistochemical staining for TH in the ventral midbrain in saline or MPTP treated mice receiving TAT or P110 at 30 days after the last MPTP or saline injection. (**B**) The total number of SNpc DA neurons and (**D**) Total striatal TH immunoreactive optical density at 30 days after the last MPTP injection. (**C**) Representative images showing striatal TH immunostaining. **Indicates p < 0.01 for saline vs. MPTP administration, and ^##^Indicates p < 0.01 for TAT vs. P110, two-way ANOVA. (n = 6 in each group). Scale Bar = 100 μm.

We next examined whether blockade of Drp1 activation by P110 affects the neurotoxicity of MPTP *in vivo*. Drp1-null mice die by embryonic day 11.5. Brain-specific Drp1 ablation causes developmental defects of the cerebellum^44^,^45^. Dopaminergic neuron specific deletion of the Drp1 gene leads to depletion of axonal mitochondria and neurodegeneration in DA neurons^46^. These findings suggest the physiological importance of Drp1 in mice. Thus, it was first necessary to determine whether P110 treatment in control mice causes any alterations in the NS (nigrostriatal) system. Because P110 specifically blocks Drp1 interaction with Fis1, which preferentially occurs under pathological condition^15^, we expected to see no difference in NS DA system under baseline conditions. Indeed, total SN TH positive neuron number and striatal TH fiber density was similar in TAT- or P110-treated saline groups (Fig. 2, saline groups), suggesting P110 treatment by itself does not affect the normal structure and function of the NS DA system.

**Figure 3:**
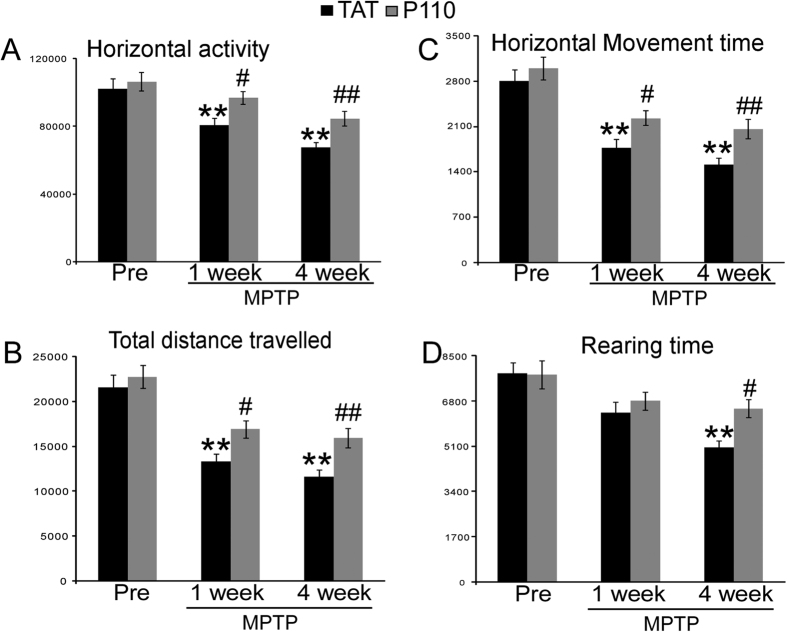
P110 treatment led to improved locomotor behaviors after P110 treatment in MPTP injected mice. (**A**) Total horizontal activity (**B**) total distance travelled (**C**) total horizontal and (**D**) Rearing time are measured in TAT or P110 treated mice before, 1 week and 4 weeks after MPTP injections. MPTP caused significant decreases in all parameters measured and P110 treatment resulted in increased locomotor functions compared to TAT treated groups. Data are mean ± SE. **Indicates p < 0.01 vs. mice injected with saline; ^#^ or ^##^Indicates p < 0.05 or p < 0.01 vs. mice treated with control peptide TAT, two-way ANOVA. (n = 12 for each group).

**Figure 4:**
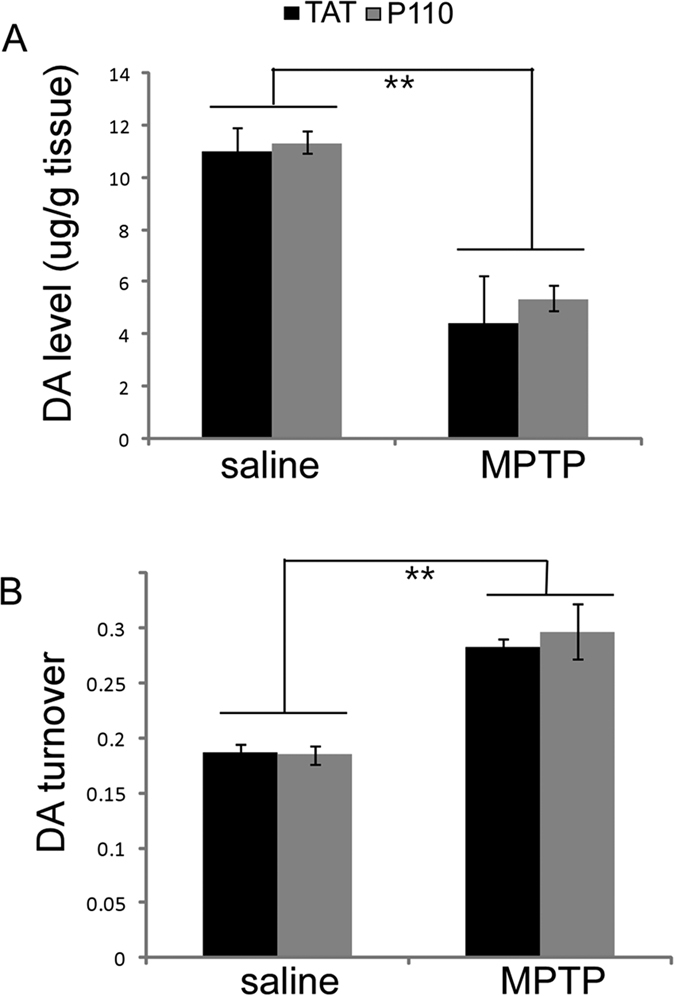
P110 treatment does not affect MPTP induced striatal DA loss and DA turnover increase in mice. Striatal DA and metabolites levels at 30 days after MPTP exposure were measured by HPLC. (**A**) DA levels were significantly decrease in MPTP injected mice both in TAT and P110 treated groups. (**B**) DA turnover is calculated as (DOPAC + HVA)/DA and MPTP exposure significantly increased DA turnover without significant differences observed between TAT and P110 treated groups. **Indicate p < 0.01 MPTP vs. saline (n = 6 for each group).

We next tested the effect of P110 treatment in mice subjected to subacute MPTP treatment. No lethality was observed in this study by the subacute MPTP injection. 30 days after the last MPTP injection, the dopaminergic system was evaluated by TH immunostaining. TH staining of cell bodies in the SN (Fig. 2A,B) and fiber densities in striatum (Fig. 2C,D) indicated that P110 treatment significantly reduced MPTP toxicity in the NS DA system. Unbiased stereology counts showed that MPTP injection significantly decreases the TH positive neurons in SN (Fig. 2A,B, Two-way ANOVA, saline vs MPTP: F_1, 22_ = 6.235, p = 0.022) and P110 treatment showed a significant protection of SN DA neurons (p = 0.001 for TAT vs. P110 within MPTP groups, ANOVA, n = 6 for each group). Similarly, quantification of TH positive fiber density in striatum decreased significantly after MPTP injections (Fig. 2C,D, two-way ANOVA, saline vs. MPTP: F_1, 22_ = 136.487, p < 0.001) and mice treated with P110 showed higher optical densities for TH immunostaining in striatum (Fig. 2C,D, p = 0.003, ANOVA, n = 6 per group). This was also manifested behaviorally, as horizontal activity, total distance traveled, horizontal movement time and vertical movement time (rearing) were all significantly decreased at 1 week and 4 weeks after MPTP injection (except for rearing activity, where there was no significant difference at 1 week but p < 0.001 at 4 weeks after MPTP injections, ANOVA, Fig. 3A–D). Treatment with P110 significantly attenuated the locomotor deficits caused by MPTP in all the parameters measured (p < 0.05 for 1 week and 4 weeks after MPTP for all parameters except rearing activity at 1 week after MPTP, ANOVA, Fig. 3A–D, n = 12 per group). Striatal DA and metabolites levels were also measured by HPLC. Our results showed that MPTP exposure resulted in significantly decreased DA levels (Fig. 4A, two-way ANOVA, saline vs. MPTP: F_1, 18_ = 44.32, p < 0.001) and increased turnover of DA (calculated as DOPAC + HVAC/DA, Fig. 4B, two-way ANOVA, saline vs. MPTP: F_1, 18_ = 34.679, p < 0.001). However, there is no significant difference between TAT vs P110 treated mice (Fig. 4, two-way ANOVA, TAT vs. P110: F_1, 18_ = 0.480, p = 0.499 for DA levels and F_1, 18_ = 0.117, p = 0.737 for DA turnover).

### Drp1 inhibition suppresses p53 mitochondria translocation and BAX and PUMA mitochondrial accumulation induced by MPTP injections

**Figure 5:**
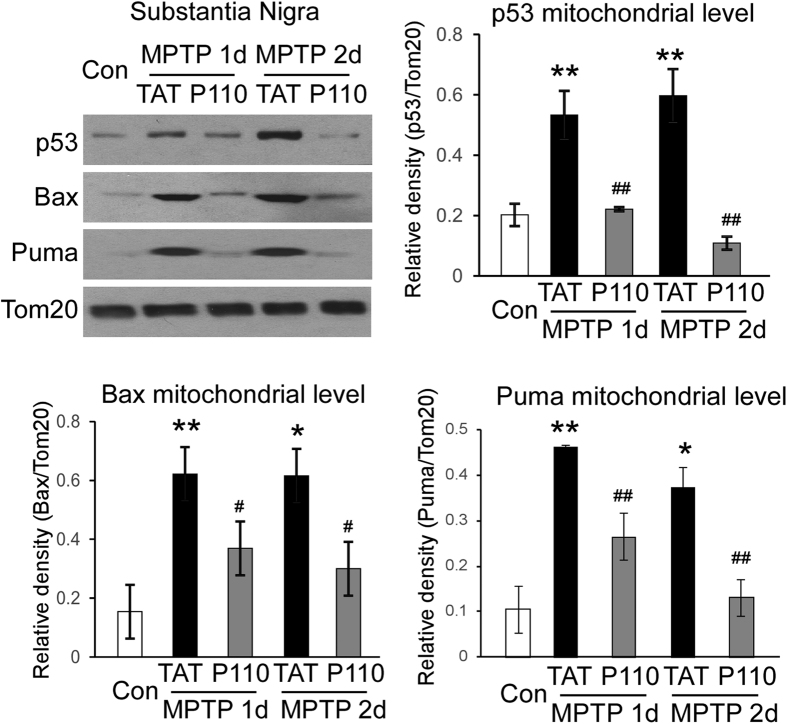
Inhibition of Drp1 hyperactivity abolished p53 translocation to mitochondria in the MPTP model and subsequent upregulation of BAX and PUMA protein levels in mitochondria. Mice were treated with the peptide P110 or control peptide and mitochondrial fractions of substantia nigra were harvested 1 and 2 days following MPTP injection. p53, Bax and PUMA protein levels were determined by Western blot analysis. Data are mean ± SE. * and **Indicate p < 0.05 or p < 0.01 vs. mice injected with saline; ^#^ or ^##^Indicates p < 0.01 or p < 0.001 vs. mice treated with control peptide TAT, two-way ANOVA. Tom20 protein levels were used as a loading control. (n = 3–4 for each group). Blots are cropped to show proteins of interest. Original blots are shown in Supplemental Fig. 4. All gels have been run under the same experimental conditions.

We have previously shown that Drp1 and p53 physically interact and Drp1 is required for p53 translocation to the mitochondria under conditions associated with brain ischemia and Huntington’s disease^23^,^24^. We next examined the mitochondrial translocation of p53 protein at SN in saline or MPTP injected mice that were treated with either TAT peptide or P110 peptide (1.5 mg/kg/day). MPTP induced p53 mitochondrial translocation both at 1 day and 2 days after MPTP injection (p < 0.05, control vs MPTP 1day or MPTP 2 day in TAT treated mice, ANOVA, Fig. 5A,B, black bars). Notably, treatment with peptide P110, which selectively inhibits Drp1 hyper-activation, abrogated this increase (p < 0.001, ANOVA, Fig. 5A,B, gray bars, n = 3–4 per group). P110 peptide amino acid sequences correspond to a short sequence within Drp1 (amino acids 49–55) and is designed to block the interaction between Drp1 and its mitochondrial adaptor Fis1^15^. We found no sequence similarity between p53 and peptide P110 or between p53 and Fis1, ruling out the possibility that P110 directly competes with p53 binding to one of these two proteins. Together, the findings are consistent with what we previously observed in the cultured cells and in other disease models, suggesting that Drp1-dependent p53 translocation to mitochondria may be a common molecular reaction to stress. Further, we found that MPTP challenge induced BAX and PUMA mitochondrial translocation in TAT treated mice (p < 0.05, Fig. 5A,C,D, black bars) and that P110 treatment abolished the BAX and PUMA mitochondrial accumulation. (Fig. 5 black bars, p < 0.01, ANOVA, n = 3–4 per group). BAX and PUMA are known p53-related mediators to trigger intrinsic apoptosis^18^,^19^. These findings thus support our hypothesis that inhibition of Drp1 hyper-activation by peptide P110 treatment suppressed pro-apoptotic signals via a p53-dependent mechanism.

### Peptide P110 treatment did not affect the early DA nerve terminal damages and subsequent activation of microglial cells and astrocytes in the MPTP-induced PD mouse model

**Figure 6:**
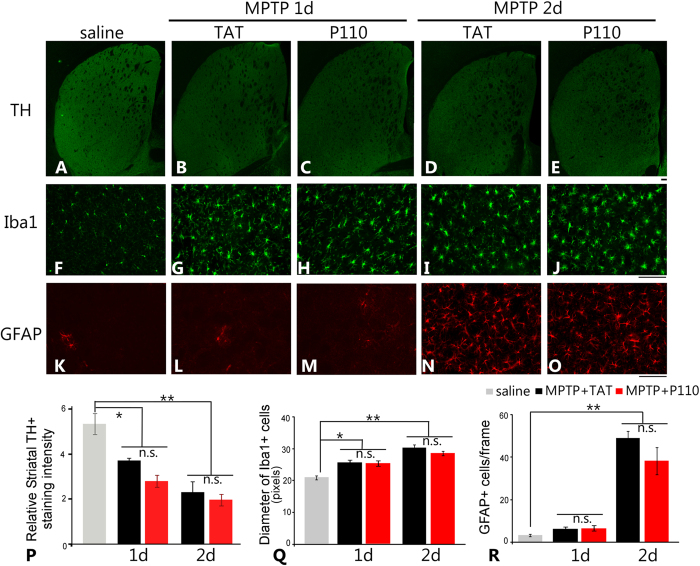
Treatment of P110 does not affect early DA nerve terminal damage and subsequent activation of microglial cells and astrocytes in the MPTP-induced PD mouse model. Striatal TH immunostaining in different treatment groups show a significant decrease in TH immunostaining intensity at both 1 day and 2 days after MPTP injection (**A–E**) and there is no difference between TAT and P110 treatment groups (quantification in panel P). Microglial activation is apparent at both 1 day and 2 days after MPTP injection (**F–J**) and no difference is observed between TAT and P110 treated mice (diameter of activated microglia cells quantified in panel Q). Astrocyte activation follows microglia activation and become apparent at 2 days after MPTP injection (**K–O**) but no difference is observed between TAT and P110 treated groups (number of GFAP + cells in striatum quantified in panel R. Data are expressed as mean ± SEM. * and **Indicates p < 0.05 or p < 0.01 versus saline group; there is no significant difference between the MPTP + TAT and MPTP + P110 groups, one-way ANOVA. Bar = 100 μm. (n = 4 in each group).

To examine whether the NS DA neuroprotection observed at 30 days after MPTP administration in P110 treated mice was due to attenuation in early dopaminergic neuronal terminal damage in the striatum by P110 treatment, we analyzed the TH fiber damage and subsequent microglial and astrocytic activation in striatum at 1 day and 2 days after the first MPTP injection in TAT or P110 treated mice. MPTP administration resulted in significant loss of TH immunostaining as early as 1 day after the first injection and sustained loss of TH fiber at 2 days post injection. (Fig. 6A–E, quantification in 6P, p < 0.05 saline vs day1 MPTP and p < 0.01 saline vs day 2 MPTP, ANOVA). Interestingly, we did not observe any difference in the early dopaminergic neuronal terminal damage in TAT and P110 treated groups (p > 0.05 for both d1 and d2 after MPTP injection, ANOVA). It has been known that the early DA neuronal terminal damage results in activation of microglial cells which subsequently induces astrogliosis in striatum^47^. In addition, astrocytic and microglical activation have been reported to be pathological features in PD^48^,^49^,^50^. Thus, we also examined whether treatment with P110 peptide inhibitor influenced the activation of astrocytes and microglial cells in the MPTP-induced PD mouse model. MPTP elicited significant microglial activation at both 1 day and 2 days after MPTP injection, indicated by enlarged cell bodies and shorter and thicker cell processes in Iba-1-labeled microglial cells (Fig. 6F–J). Consistent with previous reports, astrogliosis follows the microglial activation and was apparent at 2 days after the MPTP injection which is indicated by an increase in immuno-density of GFAP positive cells (Fig. 6K–O). Consistent with the similar early DA terminal damage in both TAT and P110 treated MPTP-injected mice, the extent of astrogliosis and microglial activation in TAT- and P110- treated mice was similar (Fig. 6). MPTP resulted in a significant enlargement in the cell bodies of Iba-1 positive microglia cells (Fig. 6Q, p < 0.05 for d1 and p < 0.01 for d2 after MPTP, ANOVA, n = 4) but there was no effect of P110 treatment in the diameter of Iba-1 positive cells (Fig. 6Q, p > 0.05 for TAT vs P110, ANOVA, n = 4) at both time points. Similarly, striatal GFAP immunostaining (Fig. 6K–O) showed robust activation of astrocytes in both TAT and P110 treated MPTP-injected mice, as demonstrated by similar cell numbers of GFAP positive cells in both groups (Fig. 6R, p > 0.05 TAT vs. P110, ANOVA, n = 4 mice for each group) at 2 days after MPTP injection. These results suggest that the neuroprotection observed at the later time point (30 days after MPTP injection) in both SN TH positive neurons and striatal TH fiber density by P110 treatment is not likely because of interference with early downregulation of TH expression, microglial activation and astrogliosis in striatum. Rather, P110 might render neuroprotection in DA neuronal cells by inhibiting p53, BAX and PUMA mitochondrial translocation, therefore blocking later apoptosis and more permanent damage of neuronal terminals of DA neurons.

## Discussion and Conclusions

This study demonstrates that inhibition of Drp1 hyper-activation by a Drp1 peptide inhibitor P110 mitigates MPTP-induced loss of dopaminergic neurons, inhibits MPTP-induced reduction in striatal dopaminergic neuronal terminal density, and attenuates the behavioral deficits induced by MPTP. Moreover, we found that inhibition of Drp1 hyperactivation mainly reduces dopaminergic neuronal injury without affecting the early activation of microglial cells and astrogliosis in the MPTP-induced PD mouse model, suggesting a selective role of Drp1 hyper-activation in the DA neuronal degeneration in PD. Furthermore, we provide evidence supporting that the neuroprotection provided by the P110 treatment might be a result of inhibition of the Drp1-dependent p53-mediated apoptotic pathway.

It has been reported that neurotoxins causing PD and PD-associated genes are related to Drp1 hyper-activation and mitochondrial fission. Dopaminergic neurotoxins induce Drp1 translocation to mitochondria and mitochondrial fragmentation^29^,^43^,^51^. PD-related proteins PTEN-induced putative kinase 1 (PINK1), parkin, DJ-1, alpha-synuclein and Leucine-rich repeat kinase 2 (LRRK2) appear to control mitochondrial function by associating with Drp1 and regulating mitochondrial fusion/fission events^52^,^53^,^54^,^55^. Inhibition of Drp1 with a Drp1 dominant negative mutant (Drp1 K38A) prevents both neurotoxin-induced and genetic PD mutant-induced mitochondrial fission abnormality and neuronal cell death in both cultured cells and Drosophila models^43^,^52^,^54^,^55^,^56^. We previously reported that inhibition of Drp1 by a Drp1 peptide inhibitor P110 reversed mitochondrial dysfunction and conferred neuroprotection in dopaminergic neurons exposed to MPP+ *in vitro*^15^. Further, we showed that P110 treatment corrected LRRK2 G2019S-induced mitochondrial dysfunction, inhibited excessive autophagy, and reduced cell death in various cell culture models, including dopaminergic neurons derived from LRRK2 G2019S PD patient-derived pluripotent stem cells (LRRK2 G2019S-iPS cells)^33^. Thus, Drp1-dependent aberrant mitochondrial fission might be a therapeutic target for treatment of PD.

The effects of Drp1 inhibition on dopaminergic neuronal cell death has not been extensively examined *in vivo*. Due to the lack of neurodegeneration in mice carrying PD-related mutant genes, in this study we utilized the widely used classic MPTP PD animal model. Noting that no PD models replicate all of the features of PD, the MPTP has become one of the most commonly used models because it produces many of the key features of PD pathology *in vivo*, especially in humans and non-human primates^57^. The subacute regimen of MPTP administration was chosen for this study because this regimen causes apoptosis and a slower onset of behavioral and DA neuronal impairment which stabilizes by 21 days after MPTP administration in contrast to the shorter time course (stable by 7 days after MPTP administration) of NS DA damage observed with the acute MPTP model^58^. The slower initiation and development of NS DA damage provides a time window allows for the evaluation of pharmacological interventions which is supported by our observation that although decreases in horizontal activities were observed as early as 1 week after MPTP injection, the vertical activity deficits were not significant at 1 week but were significant at 4 weeks after MPTP injection (Fig. 3). Consistently, P110 treatment did not alter vertical activity at 1 week post MPTP injections but significantly improved both horizontal and vertical activities at the 4 week time point (Fig. 3D). To examine whether P110 treatment improved the locomotor activity in MPTP treated mice by increasing DA levels in the striatum, we also measured DA levels and DA turnover in saline or MPTP injected mice that received TAT or P110 treatment. Our data showed that although DA neurons were protected by P110 treatment (Fig. 2) and P110 treated mice showed improved behavioral measurements (Fig. 3), DA levels in striatum at 30 days after MPTP exposure were not significantly different in TAT or P110 treated groups (Fig. 4). There are multiple possible explanations for the improved locomotor behavioral measurements in the absence of changes in total striatal DA levels. First, it is possible that the behavioral improvement in P110 treated mice might not be related to the midbrain DA system but rather through protection against MPTP toxicity in the peripheral system. Second, most dopamine (80–85%) in DA terminals is in the storage granules which are reserpine-sensitive. Dopamine released for neurotransmission is thought to be largely from the newly synthesized pool, which is sensitive to inhibition by alpha methyl paratyrosine. Thus, in many paradigms DA transmission is altered while DA levels are little changed^59^. Lastly, our previous study has shown that P110 treatment can affect striatal medium-sized spiny neurons (MSNs)^24^. Therefore, it is possible that P110 treatment enhanced the locomotor activity in MPTP treated mice through alterations of postsynaptic signals without affecting the total DA levels in striatum. The precise mechanisms of P110 on the improvement of locomotor function in MPTP treated mice thus warrants further investigation in future studies.

Mitochondrial fission is thought to be balanced by mitochondrial fusion to regulate mitochondrial number, morphology and distribution, the process of which is critical for maintaining mitochondrial bioenergetic activity and function. Loss of fusion protein mitofusin 2 (Mfn2) in DA neurons has been shown to produce severe respiratory chain deficiency and lead to the loss of DA nerve terminals in striatum^60^, suggesting that mitochondrial fusion is required for axon development of DA neurons. Moreover, mutations in PINK1 and Parkin can accelerate Mfns1/2 degradation which results in mitochondrial fragmentation in Drosophila and human SH-SY5Y cells^61^,^62^,^63^. It will be of importance to determine whether mitochondrial fusion is impaired in the PD mouse model and how the fusion and fission proteins interact to regulate DA neuronal survival in PD animal models.

Mff, MiD49/51 and Fis1 have so far been identified as adaptors of Drp1 on mitochondria in mammals^64^,^65^,^66^. Otera. *et al*. reported that Drp1 can be recruited by Mff to induce mitochondrial fission without the requirement of Fis1 function^64^. We previously showed that Drp1 binding to Fis1 mainly occurs under stress while much weaker binding of Drp1/Fis1 is observed under normal conditions^15^,^34^. Thus, these fission adaptors may play different roles in baseline mitochondrial fission and mitochondrial fission occurring under pathological conditions. P110 was designed based on a rational approach to interfere with the interaction between Drp1 and Fis1^15^. We reported that blocking Drp1/Fis1 interaction by P110 treatment abolished Drp1 translocation to mitochondria, reduced mitochondrial fragmentation, suppressed mitochondrial depolarization and oxidative stress, corrected ATP content and reduced cell death in a number of disease models *in vitro* and in disease models *in vivo* other than PD^15^,^23^,^24^,^33^,^34^. Importantly, in normal cells P110 had minimal effects on Drp1 mitochondrial levels, mitochondrial morphology, mitochondrial function and cell survival^15^,^23^,^24^,^33^,^34^. P110 did not influence mitochondrial fusion-related proteins including Mitofusin 1 and 2 and OPA1, and other mitochondrial fission adaptors such as Mff and Mid49/51^15^,^33^. P110 can enter brains and its biological effects require the presence of Drp1; the loss of Drp1 either by RNA interference (siRNA) or in Drp1 knock-out MEF cells completely abolished the protection by P110^23^,^24^. These findings further support the specificity and selectivity of P110. In the current study, using the MPTP-induced PD mouse model, we consistently observed that treatment with P110 inhibited enhanced Drp1 translocation to mitochondria induced by MPTP injection while it has minimal effects on Drp1 mitochondrial levels and DA neuronal number and structure in normal mice treated with saline. These findings are consistent with our previous observations that P110 treatment has little effect on mitochondrial and neuronal functions at the physiological level^15^,^23^,^24^,^33^,^34^, which is likely the results of less binding of Drp1/Fis1 under normal conditions. In contrast, we observed significant neuroprotection from P110 treatment in mice subjected to MPTP injections.

Microglial activation and reactive astrogliosis are characteristic key features of many CNS injuries including the MPTP PD models. In our study, at 24 and 48 hours after the first MPTP injection, when DA nerve terminal is damaged while no apparent DA neuronal loss is present as yet, we observed significant microglial activation demonstrated by enlargement of the cell body and shortened cellular processes in microglial cells as well as reactive astrogliosis at 48 hours post MPTP injection identified by GFAP positive astrocytes in the striatum in both TAT control peptide and P110 peptide treated MPTP-injected mice (Fig. 6). Previous studies have shown that dopaminergic neuronal terminal damage causes microglial activation and subsequent astrocyte activation through the JAK2-STA3 signaling pathway^67^. DA terminal damage appears to be required for the activation of microglia and astrocytes because both inhibition of MPTP to MPP+ conversion^47^ and inhibition of MPP+ uptake into dopaminergic neurons^67^ abolish dopaminergic terminal damage and astrogliosis. In this study, MPTP and MPP+ levels were not measured in TAT or P110 treated mice, however, the P110 treatment is not likely to affect the metabolism of MPTP since the loss of TH in striatum at early time points and subsequent microglial and astrocytic activation were similar in TAT and P110 treated groups (Fig. 6). Instead, it might inhibit the later apoptotic pathway activation through the Drp1-p53 pathway which resulted in preservation of DA neurons in SN. This hypothesis is consistent with our observation that the protection of DA neurons in SN is greater in P110 treated mice compared to the effects of this treatment on the preservation of striatal TH fiber density. At least two interesting questions remain to be answered in future studies. Will longer treatment times and longer recovery times allow regeneration or repair of the DA neuronal terminals in striatum in P110 treated mice? Similarly it is important to test whether delayed treatment with P110 peptide at different time points after the initiation of MPTP injections would be effective in protecting the NS dopaminergic system. Although P110 treatment was able to protect DA neurons in the SN without affecting early microglial activation and reactive astrogliosis, our data also showed that the protection of striatal DA nerve terminal is only partial. Since a potential contribution of neuro-inflammation to DA terminal damage has been suggested by previous studies^68^,^69^; a combined treatment with P110 and inhibitors of neuro-inflammation might be needed to provide a more robust protection of both SN and Striatal DA nerve terminals in the development of a potential PD therapy.

In summary, our studies provide evidence for the importance of Drp1 translocation to mitochondria in dopaminergic cell death in PD. Our findings suggest that inhibiting Drp1 hyperactivation, such as by P110, may be a useful strategy to reduce PD-associated pathologies.

## Additional Information

**How to cite this article**: Filichia, E. *et al*. Inhibition of Drp1 mitochondrial translocation provides neural protection in dopaminergic system in a Parkinson’s disease model induced by MPTP. *Sci. Rep.* **6**, 32656; doi: 10.1038/srep32656 (2016).

## Supplementary Material

Supplementary Information
