# Supplementary material for: Inhibition of Drp1 mitochondrial translocation provides neural protection in dopaminergic system in a Parkinson’s disease model induced by MPTP
Source: Sci Rep. 2016 Sep 13;6:32656. doi: 10.1038/srep32656 (PMC5020318; doi:10.1038/srep32656)
Supplement: Supplementary Information [file srep32656-s1.doc]

**Inhibition of Drp1 mitochondrial translocation provides neural protection in dopaminergic system in a Parkinson’s disease model induced by MPTP**

Emily Filichia^1^, BA, Barry Hoffer^1^, MD PhD, Xin Qi^2*^, PhD, Yu Luo^1*^, PhD

^1^Department of Neurological Surgery, Case Western Reserve University, Cleveland, USA

^2^Department of Physiology & Biophysics, Case Western Reserve University, Cleveland, USA

Correspondence to

*Yu Luo, PhD, Department of Neurological Surgery, Case Western Reserve University, 2109 Adelbert Rd, Cleveland, OH, USA. Email: [yxl710@case.edu](mailto:yxl710@case.edu), Phone: 01-216-368-4169,

*Xin Qi, PhD, Department of Physiology & Biophysics, Case Western Reserve University, Cleveland, USA, Email: [xxq38@case.edu](mailto:xxq38@case.edu), Phone: 216-368-4459

Supplementary Figures:


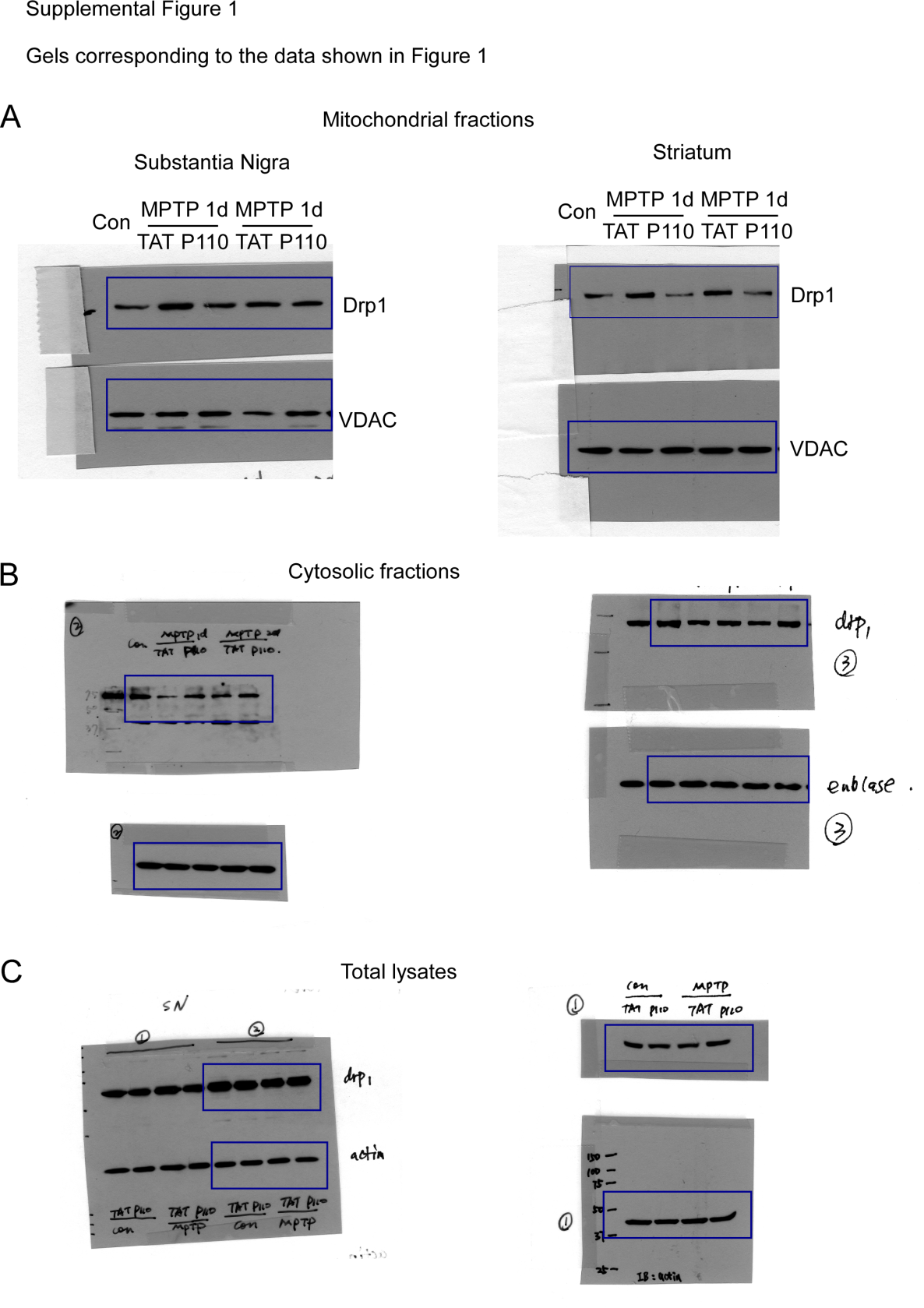


Supplementary Figure 1. Original Western blots of Fig 1. All gels have been run under the same experimental conditions.


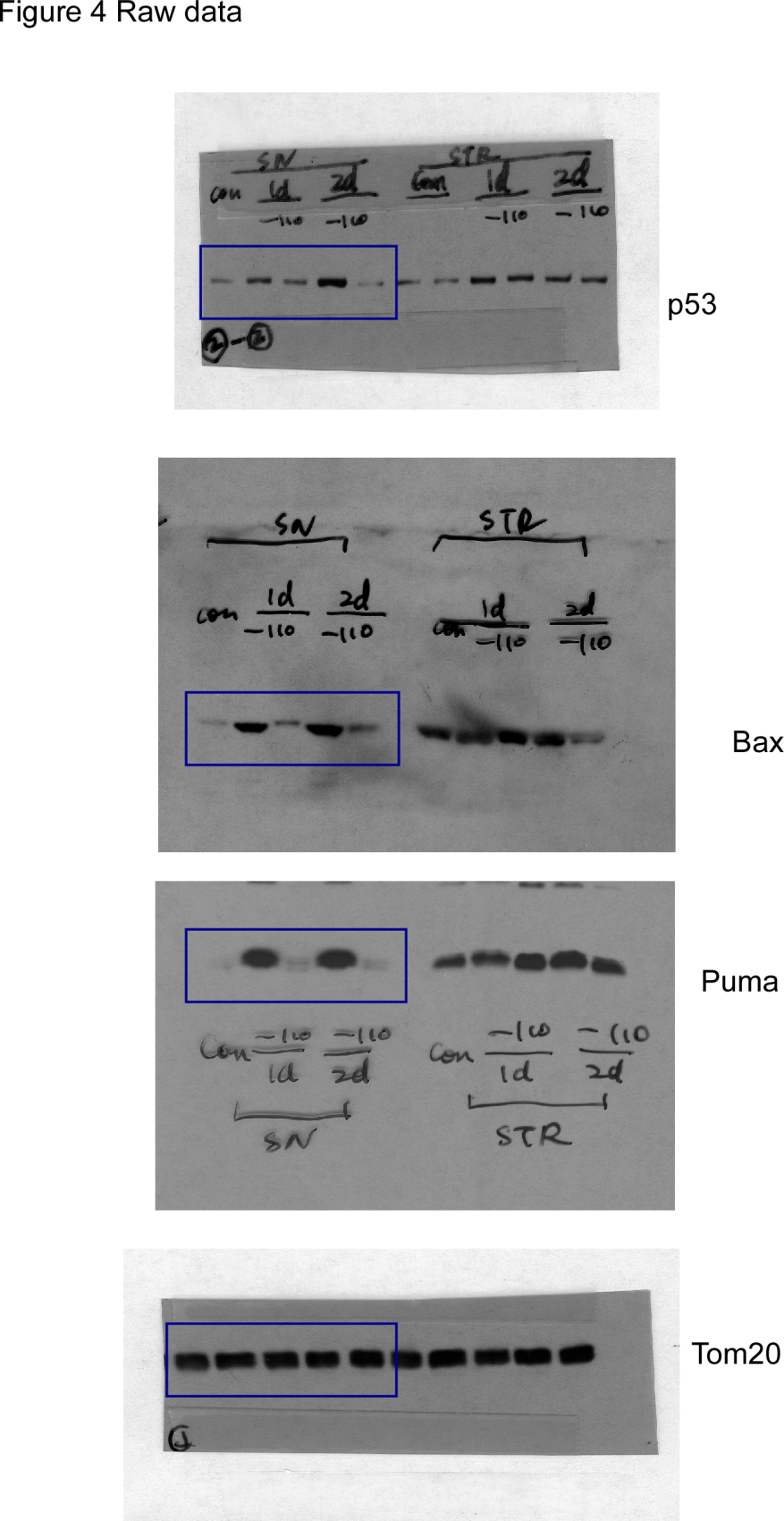


Supplementary Figure 2. Original Western blots of Fig 5. All gels have been run under the same experimental conditions.
